# Adult patient perspectives on receiving hospital discharge letters: a corpus analysis of patient interviews

**DOI:** 10.1186/s12913-020-05250-1

**Published:** 2020-06-15

**Authors:** Katharine Weetman, Jeremy Dale, Emma Scott, Stephanie Schnurr

**Affiliations:** 1 Unit of Academic Primary Care, Warwick Medical School, University of Warwick, Coventry, CV4 7AL UK; 2 Centre for Applied Linguistics, University of Warwick, Coventry, UK

**Keywords:** Patient discharge summaries, Patient education, Copy letters, Discharge letters, Doctor and patient communication, Hospital discharge, Applied linguistics, Discharge communication

## Abstract

**Background:**

UK government guidelines and initiatives emphasise equity in delivery of care, shared decision-making, and patient-centred care. This includes sharing information with patients as partners in health decisions and empowering them to manage their health effectively. In the UK, general practitioners (GPs) routinely receive hospital discharge letters; while patients receiving copies of such letters is seen as “good practice” and recommended, it is not standardised. The effects and consequences of whether or not this happens remains unclear. The aim of this study (one of three forming the Discharge Communication Study) was to explore patient perspectives on receiving discharge letters and their views on how this could be improved in order to optimise patient experience and outcomes.

**Methods:**

Semi-structured interviews were conducted with a diverse sample of 50 patients recruited from 17 GP surgeries within the West Midlands, UK. All participants were adults with a recent episode of general hospital inpatient or outpatient care. Data were audio recorded, transcribed and analysed using mixed methods corpus linguistics techniques.

**Results:**

Participants reported inconsistent access to discharge letters. Most wanted to receive a copy of their discharge letter although some expressed reservations. Perceived benefits included: increased understanding of their condition and treatment, reduced anxiety, and increased satisfaction. Consequences where participants had not received letters included: letter inaccuracies being overlooked, missed follow up actions, failure to fully remember diagnosis, treatment, or self-management or recommendations, and confusion and anxiety at what occurred and what will happen next. Participants felt the usefulness of receiving copies of letters could be increased by: including a patient information section, avoidance of acronyms, and jargon or technical terms explained with lay language.

**Conclusions:**

Most patients value receiving copies of hospital discharge letters, and should be consistently offered them. Patients’ preferences for letter receipt could be logged in their health records. To enable positive outcomes letters should have a clear and accessible format that reflects the priorities and information needs of patients. Patients appear not to be receiving or being offered copies of letters consistently despite UK policies and guidelines supporting this practice; this suggests a need for greater standardisation of practice.

## Background

UK government guidelines and initiatives [1–3] emphasise equity in delivery of care, shared decision-making, and patient-centred care. This includes sharing information with patients as partners in health decisions and empowering them to manage their health effectively. The recent “please write to me” [4] initiative by the Academy of Medical Royal Colleges sought to increase practice of patients receiving letters through encouraging clinicians to write to patients directly in plain language and copy the letters to the patient’s General Practitioner (GP); this initiative [4] has received both support [5] and criticism [6]. In the UK, GPs routinely receive hospital discharge letters whilst patients may or not be copied into this correspondence [7, 8].

Although copying letters to patients is currently considered to be good practice in the UK [1–4], it is not standardised. The effects and consequences of this on patient outcomes remains unclear [9, 10]. Internationally, the practice of patients receiving letters varies, but as Rayner et al. [11] summarise, in many countries it is common practice for hospital doctors to write directly to GPs (or equivalent) and for patients to receive copies [11]. Research in some countries, such as Australia [12, 13] and the Netherlands [14], has looked at the use of patient-directed or personalised letters.

Despite past studies reporting high rates of patient preference for receiving letters [15–24], previous literature has reported inconsistency of this practice [7, 8]. Prior to this study we conducted a realist review [10] relating to this topic which found that whilst there is research and guidance that argues for the benefits of copies of discharge letters, there is little evidence about the specific contexts in which this may or may not be helpful [9, 10]. Another review on patient letters, by Harris et al. [9] in 2018, concluded, “*evidence to support the benefits of copy letter practice as described in health policy remains unclear”* and that, *“little is known about the impact of copy letters on health outcomes”* [9] (p.2080).

This study was conducted as part of a PhD and formed one of three studies [25] which sought to explore the contexts relating to how patients receiving discharge letters may work or not from the perspectives of patients, GPs [26] and hospital clinicians respectively (see the Discharge Communication Study [25])*.* This study focussed on the perspectives of patients and involved in depth interviews with a sample of recently discharged patients in order to answer the following research questions (RQs):

1. According to patients, in what form do they currently receive discharge letters and why?
2. According to patients, should they receive or not receive discharge letters, why and in what form?

## Methods

The study opened in October 2017. GPs at 18 participating practices across a diverse range of settings took part in sampling discharge letters. The practices were spread across urban, semi-urban and rural settings in the West Midlands of England, including the regions of Coventry, Rugby, Herefordshire, and Warwickshire. These areas have varying socioeconomic characteristics. For example, South Warwickshire is relatively affluent with a predominantly white ethnic population whereas Coventry has a more ethnically diverse population and includes neighbourhoods which are amongst the most deprived in England [27–29].

GPs were asked to screen (see Table 1 for screening criteria) and select a sample of recently received discharge letters according to what they considered to be “successful” or “unsuccessful” letter exemplars. As detailed in the published research protocol for the Discharge Communication Study [25], there were no set criteria for letter categorisation; instead, the intention was to learn from each participating GP’s interpretation of what makes a “successful” or “unsuccessful” discharge letter. Thus, letter sampling was purposive [30–32]. This approach was intended to increase sample diversity and address the research questions within dichotomous contexts.

**Table 1:** Selection criteria for participating patients

Inclusion criteria	• Adult (18+ years) patients recently (≤3 weeks) discharged from a hospital following an episode of inpatient or outpatient care. • Registered with participating GP practice. • Treated at and discharged from a hospital within North or South Warwickshire, Coventry, Rugby, Herefordshire or Worcestershire. • Written discharge communication has been sent to the patient’s GP.
Exclusion criteria	• Age < 18 years. • Lack capacity to give informed consent to participate in the study (e.g. Alzheimer’s, severe mental illness etc.) or are deemed by the GP to be unsuitable for participation (e.g. end of life). • Discharged to providers or units other than their GP (e.g. discharge from hospital to a rehab unit). • Discharge communication from mental health services. • Communication about individuals who are considered unable to participate in an interview conducted in English. • Have expressed a general wish not to participate in research.

The patients associated with each of these letters were then sent an invitation pack by their GP practice which asked those interested in participating to contact the research team directly to arrange an interview. The intention was for patients to participate within 4–6 weeks from the date of their recent episode of hospital care. The invitation materials for patients stated that they had the option to enquire about viewing a copy of their recent discharge letter had they not done so previously; this was enabled at interview if their GP was in agreement [25]. For those who had received a copy of their discharge letter, they were encouraged to bring or have it available at the interview.

Interviews took place with KW at GP surgeries or at participants’ homes. Interviews were “semi-structured” [33] with eight predominantly open questions based on discharge communication (interview guide in Additional file 1). The questions explored patients’ most recent discharge experiences, their preferences and desired formats for receiving discharge letters (or not), and their suggestions for improving discharge communications. Interviews were digitally recorded and transcribed by KW. Interviews ranged from approximately 10–90 minutes and on average took around 60 minutes.

Corpus Linguistics (CL) was identified as a suitable analytical technique due to the potential to rapidly analyse a large qualitative database whilst still providing scope for in-depth analyses [34, 35]. A “corpus” may be defined as *“a body of written text or transcribed speech which can serve as a basis for linguistic analysis and description”* (p.12) [36]. CL focuses on analysing patterns of co-occurrence and meanings in data. Corpus processing can reveal language patterns and commonalities as well as rare cases; neither of which are likely to be reliably available through manual searching or intuition alone [37–39]. CL has successfully been employed for health-focussed research [40–45].

Interviews were audio recorded and transcribed by KW to build the corpus in *Antconc* [46]. CL analysis of the corpus involved quantitative techniques via *keywords* for initial pattern identification [47] and expounding salient corpus features [47]; Adolphs et al. [48] describe this approach as *“taking the pulse”* (p.25) [48] of the data. *Keywords* are generated when one corpus is statistically compared with another [35]; this provides information about the *“keyness or specificity of a given corpus in terms of what it is about” (p.25)* [49]. The BNC Spoken (2014) [50] was used as a reference corpus for generation of keywords. For this research, log-likelihood (*p* < .05) was used to calculate and statistically analyse significance within the corpus for the top 100 keywords. Keyword analysis and quantification of language features within interview speech was considered useful to ground the analysis because, as suggested in previous CL literature [51], this reduced the bias of selecting words which were seemingly important and then establishing their importance, instead, quantitative CL techniques allowed words and features of interviews to be revealed which may have otherwise been “missed” or not known to be of importance. Hence, keyword findings grounded and guided the qualitative analysis that followed.

Qualitative techniques were undertaken by KW, specifically, *collocation* (4 word span) and *concordance* line inspection for expansion of quantitative findings [47]. *Collocates* are words that statistically significantly co-occur with one another [52, 53]. Concordance lines are whereby words of interest, or “nodes” (e.g. keywords) are displayed with a defined span of co-text either side in order to examine the word in context [35]. Concordance outputs were “lemmatised” [54] and so encompassed all variants of a word *lemma* or base (e.g. to discharge) to include all inflectional forms such as those marked for different person or tense (e.g. discharged, discharging...). During analyses, observations were also made in regard to *dispersion* [55, 56]. As the interview questions (Qs) were designed around the RQs, consolidated responses for each interview question were analysed in turn. The entire corpus was then hand searched by KW for any missed patterns in order to increase reliability and validity of findings [57] and attain or at least come close to attaining *pattern saturation* [45, 58] for the RQs. Following initial analysis by KW, results and analyses were then reviewed and iteratively refined through discussion with other members of the research team (ES, JD, SS).

## Results

### Recruitment and data collection

A total of 50 patients from 17 of the GP practices participated. The median number of interviews per GP practice was two; range 1–10. Thirty-two (64%) patients reported that they had received a copy of the discharge letter from the hospital; 28 of these patients had the letter with them for the interview. Fifteen patients were shown their letter for the first time at interview. In total, 43 had their discharge letter at interview. Several participants attended the interview with a partner or relative. Partners and relatives predominantly provided practical and emotional support and aided memory recall. Those who wanted to formally participate in interviews signed consent forms; this occurred for five interviews. Some partners and relatives talked during interviews but did not sign consent forms and so their contributions were not transcribed.

The table in Additional file 2 summarises the patient characteristics in terms of demographics and the specialty associated with the discharge letter. Notably, it was optional for demographics to be self-reported as part of the consent form using open format questions and hence data were only available for those participants who provided this information.

Most (88%) of the participant's discharge letters related to recent inpatient admissions. The letters covered 15 specialties with the highest numbers of cases from Trauma and Orthopaedics (*N* = 8, 16%) and General Surgery (*N* = 7, 14%). Participant ages ranged from 27 to 87 years (median = 64). Within the patient participant sample, 64% related to “successful” GP-graded letters and 36% related to “unsuccessful” GP-graded letters.

### Corpus linguistic analysis

The corpus contained eight text files (one per interview question response) for each of the 50 participants; this totalled 400 text files. Partner or relative contributions were removed prior to analysis to focus the corpus on patients’ perspectives. Following initial corpus analyses, partner and relative contributions were separately hand-searched for additional patterns or meanings but none were identified.

The total number of “words” or tokens [53] was 135,637 with 4402 “word types” or token types [53]. Text file lengths for interviews ranged considerably (256–5978), with a median interview length of 2622 words. The top 100 keywords for the entire corpus, calculated by log-likelihood (*p* < .05), are in Additional file 3. Within the sections below, verbatim quotes are used to illustrate patterns identified through analyses. Where a longer co-text is required to contextualise lines and findings, run-on quotations have been used. Samples of keyword concordances to support study findings and result observations are in Additional file 4. Table 2 summarises the keyword results for the corpus and sub-corpora in respect of the RQs (see Additional file 5 for table of word counts for the entire patient interview corpus and sub-corpora – collated responses for each interview question).

**Table 2:** Keyword groupings relevant to research questions by question and full corpus

Interview question (Q)	RQ relevance	Keywords relevant to RQ(s) and Qs
Q1: Please tell me about your experiences of receiving any form of written discharge communication?	RQ1	Discharge(d), hospital, letter, doctor(s), medication, GP, information, nurse, tablets, surgery, received, telephoned, given, copy, consultant, papers, appointment, operation, sent, patient
Q2: When you were discharged from hospital recently, what information were your given?	RQ1	Discharge(d), hospital, letter, information, doctor(s), medication(s), given, GP, consultant, summary, papers, said, told, communication, aftercare, follow
Q3: How did you feel about the information you were given?	RQ1/RQ2	Hospital, discharge(d), letter, information, doctor, GP, medication, reads, given, patient, medical, told, summary
Q4: What written information would you like to be given or sent when being discharged from hospital and why?	RQ2	Hospital, information, discharge(d), letter, know, medication, given, patient(s), summary, think, medical
Q5: Would you prefer to receive a direct copy of the letter sent to your GP or a separate letter specifically addressed to yourself?	RQ2	GP(s), letter, copy, information, discharge(d), patient, understand, personalised, medical
Q6: Would you like to always be given a letter or would you prefer to choose each time you are discharged?	RQ2	Letter(s), discharge(d), GP, information, patient(s), think, opt, copy, given, time, medical, automatic, system
Q7: How do you think the process of patients receiving written discharge communication can be improved?	RQ1/RQ2	Discharge(d), information, letter, patient(s), communication, waiting, given, copy, time
Q8: Is there anything else you would like to talk to me about today related to written discharge communication?	RQ1/RQ2	Hospital, patient(s), discharge(d), think, letter, communication, experience, information, medical, NHS
Full patient corpus (all questions compiled)	RQ1/RQ2	Hospital, discharge(d), letter(s), GP, information, doctor(s), patient(s), medication, given, know, surgery, think, copy, medical, communication, summary, follow, aftercare, time, told, tablets, treatment, understand, results, tests, waiting, paperwork, anything, received, blood, read, happened

#### Q1 - Please tell me about your experiences of receiving any form of written discharge communication?

Several “discharge” lines (*N* = 146) were explicit evaluations of the discharge communication quality. Cases where participants had been “given” written information, so long as it was described as accurate and appropriate, tended to co-occur with positive evaluations such as *“brilliant”, “handy”, “comprehensive”.* Conversely, lines where participants had not been given adequate written information or where this information was inaccurate tended to be peppered with negative evaluations such as *“incorrect”, “just [GIVE]”* and *“wrong”.* However, exceptions were found and some participants felt discharge letters in general were not useful. Reasons for this included the density of unexplained medical terminology and the perceived poor quality of communications. Other cases framed as negative were instances where the participant was requested to deliver the letter to the GP:

*“I think when you are on your back and not feeling too good after being discharged the last thing you want to do is go to see your GP … ”*

Within the sample, few participants (*N* = 2, 4%) reported having experience of receiving a personalised discharge letter as opposed to a GP copy; these related to inpatient letters from medical specialties. Participant evaluations of such letters were unmistakably negative, *“minimal in information”, “no real useful information”*. One participant compared the personalised letter against the GP letter, “*… I really don’t see what use that is to me as a patient (.) whereas the letter to the General Practitioner is totally different …*”.

Many “discharge” lines were statements of letter receipt (or not) (e.g. *“I received a um discharge summary”*). Some participants noted inconsistencies within their own experiences and one participant directly reflected on this and told how they looked up discharge policies prior to interview and was surprised there is not a standardised policy:

*“When I googled the website yesterday about discharging from NHS hospitals and it says each hospital has its own discharge policy I am just wondering why (.) Why isn’t there a standard discharge policy? Why does each hospital have a different one?”*

Throughout “discharge” lines, participants emphasised *timing*; this included discharge wait times, and time-frames in receiving discharge letters and medication (see Table 3). Hand-searching [DISCHARGE] concordance lines for concepts of timing indicated that time frames were perceived by participants to be unnecessarily long. This was conveyed through items in the co-text with negative connotations such as *“hanging around”* or *“worst part”* as well as phrases that communicated the *timing* was of an unnecessary or unpleasant duration *“more than”* and “*nearly* [X time]” (see Table 3).

**Table 3:** Sample of 10 lines for [DISCHARGE] in Q1 relating to concept of timing

it was affecting my kidney I was	**discharged**	after five hours of waiting with the person
one it was (.) not that I was	**discharged**	very quickly because I was out of the
I say to get the medication and	**discharge**	letter it was more than twenty four hours
3 weeks from the date I was	**discharged**	uh before the surgery received any
hanging around to wait for these	**discharge**	papers and that’s what I was waiting
a whole day in hospital for that	**discharge**	letter to come from the doctors and my
little writing (.) yeah [DATE] they	**discharged**	me and I didn’t have to hang
(.) to be fair when you get	**discharged**	from hospital all you are interested in is
to wait nearly 9 hours for that	**discharge**	letter so that was probably the worst part
going to be ready where is the	**discharge**	letter because we didn’t know what the

#### Q2 – When you were discharged [recently], what information were you given?

Results for “information” for Q2 (*N* = 37) demonstrated that participants often described specific details which they conceptualised as important. These included: medication information, PALS team information, and information relating to treatment and tests, as well as care management, results, and follow up.

Although receiving letters may provide an opportunity to correct misunderstandings or inaccuracies, in certain cases, the presence of a discrepancy itself can cause harm. One participant explained how the letter wording in relation to alcohol consumption felt accusatory, *“makes me sound awful” “I just think it’s not fair”* as they did not believe the facts had been accurately relayed. Although patients do not tend to mind reference to social habits in letters as long as they are relevant [59], the above case caveats this notion in that sensitive information should be cautiously fact-checked and presented using neutral, non-judgemental language.

#### Q3 – How did you feel about the information you were given?

Typical uses of [FEEL] (*N* = 94) in the Q3 sub-corpus were references to participants’ internal emotions or psychological responses and perceptions (see Table 4 sample).

**Table 4:** Sample of 10 lines for lemma [FEEL] in Q3 from 10 different participants

recommend you should do (.) I did	**feel**	a bit uneasy (.) they had obviously ruled out
medication or review so that’s where I	**feel**	there was an error definitely (.) so it wasn’t
well so it just wasn’t organised and I did	**feel**	like they just completely wiped their hands
so I know no it was a good summary I	**felt**	it was a good summary (.) that I could
it (.) it won’t go nowhere (.) but I	**felt**	very strongly this time that the (.) it was very
feeling uh I think it adds to your	**feeling**	of vulnerability because you can’t have a full
go through my mind of you know	**feeling**	worried about going under you know so it
plates in my leg at the time so I	**feel**	that process and the interaction has
I did I felt alright about it um I	**felt**	a bit that why did they put this instead of
positive step forward I definitely	**felt**	good about it yeah and there was some trust

One participant felt that for them, *“the knowing is better than the not knowing”* and described the confusion of not knowing negatively, *“nightmare”*. For this participant, the *“knowing”* through receiving a letter improved not only their experience and knowledge but positively impact their wellbeing, by reducing confusion, *“it was so much less stressful”*.

Across [FEEL] lines, a theme emerged of *confusion*. This ranged from “medication” plans to confusion regarding physical rehabilitation (e.g. exercise regime, time off work …), whether or not there would be follow up (and what), and how pending investigations and results would be arranged and/or communicated. In several instances, confusion stemmed from “medical” information in the letter which was dense with jargon or otherwise incomprehensible to participants, *“it went a bit over me head”*. Some participants suggested solutions, such as providing the letters alongside verbal advice, looking up terms on the internet, or suggesting that letters could contain lay explanations for terminology or jargon.

#### Q4 – What written information would you like to be given or sent when being discharged from hospital and why?

Hand-searching uncovered that within the study sample, most participants (44/50) wanted to receive a discharge letter. Written information was often framed as a “need” as opposed to merely a “want”. For four participants this “need” was explicit (e.g. “*you need to have the information in the discharge”*). Within other lines, the “need” for information was more implicit and conveyed through indicative phrases (e.g. *“important”, “anything else is … not worth talking about”)*. One participant drew on the field of basic human needs to suggest they should not be *“starved”* of written information. Additionally, a few participants described how receiving letters can allow patients to correct inaccuracies, and that this can improve the record quality and prevent adverse outcomes.

Some patients suggested they may not want to receive bad news letters. However, these cases were hypothetical and the few patients in the study who had received bad news or serious diagnoses reported to find the copy letter useful and informative. Generally, patients’ desires for written discharge information pervaded all types of discharge episodes.

#### Q5 – Would you prefer to receive a direct copy of the letter sent to your GP or a separate letter specifically addressed to yourself?

Hand-searching found: 31 (62%) participants preferred to receive a direct GP copy letter, 9 (18%) had no preference, 7 (14%) wanted a patient personalised letter, 2 (4%) wanted to receive both forms, and 1 (2%) wanted neither. “Personalised” lines (*N* = 8) contained mixed viewpoints; one participant said this would be *“best”,* several appeared indifferent *“I don’t mind”,* and one was against the practice, *“it would be going too far”*. “Copy” lines (*N* = 36) seemed to show preference for “copy” letters, (e.g. *“I think yes we should have a copy of things sent to the doctor”*) although responses were not always strongly weighted; some simply said that a “copy” would be *“fine”*. Reasons for “copy” form preference varied. One participant felt copy letters should be received as information should not be *“hidden from you”.* Another participant rationalised that the GP and patient having the same discharge “copy” simplifies communications and justifies that to have different discharge forms would *“defeat the object”*. Several participants explained that having a copy of the GP letter can help keep them informed and act as a reference for future consultations.

A few participants queried whether patient letters are for bureaucratic purposes, *“it just seems to me out of bureaucracy you send a copy to the GP and send a copy to the patient (.) and I’m not too sure that’s necessary”.* This is where *patient choice* becomes instrumental to policies so that patients are not needlessly blanket-copied into all letters.

#### Q6 – Would you like to always be given this letter or would you prefer to choose each time you are discharged?

Hand-searching found 44/50 (88%) participants would always like letters (e.g. *“Um I think probably all the time I would want to know what’s been going on”*) and 6/50 (12%) preferred to choose (e.g. *“uh (.) probably choose each time you are discharged actually (.)”*).

Reasons for “opt in” preference tended to include making sure there is no waste and ensuring that letters are only received by those who want them. Reasons for “opt out” were that it should be automatic and that it is the patient’s right to view information as it concerns “*[their] body”*. One participant noted that this system could reduce inadvertent errors:

*“I think there is probably less room for errors and mistakes if it’s the norm (.) rather than if it’s the addition”*

Lines for the lemma “automatic” (*N* = 15, 10 texts) reinforced favourability for an “opt out” style system due to its potential for consistency. Within “automatic” lines, patients articulated that they should not be burdened with asking each discharge, *“I think if you are not feeling well you don’t want to make decisions about stuff do you”*. Reflections on the wider public opinion were that only a minority of people would not want to receive letters; this was indicated through low level quantifying language, *“some”, “fewer”.*

#### Q7 – How do you think the process of patients receiving written discharge communication can be improved?

Participants articulated that greater consistency relating to this practice is required:

*“it should be a (.) should not a guidelines or we recommend this should happen (.) because there isn’t no negative to it is there if the patient don’t want it then they don’t have to read it so there is only going to be a positive”*

One participant suggested letter receipt should be recorded on the hospital system:

*“I think that it should be a part of their discharge to say did patient have a copy (.) because then you would be able to audit really so even if it was like a yes or no box”*

Another participant suggested that providing patients with discharge letters can increase their autonomy and encourage them to *“take ownership”*. Participants argued that receiving letters means they can assist with actionable components to ensure nothing is missed.

Several participants felt email may be superior and timelier to paper letters:

*“so if it could be electronically as we said through email that would be fantastic I think it would speed up the time and free beds and free time for the doctor”*

#### Q8 – Is there anything else you would like to talk to [the interviewer] about today related to written discharge communication?

Participants conveyed that discharge communications needs improvement (e.g. *“there is not enough communication between the hospital and doctors”*). Additionally, participants commented on the variation of receiving letters and considered possible benefits of standardised practices, *“they should give all the details to the patient in the discharge letter at least”*. One participant summarised that poor written discharge communication with patients leads to adverse outcomes like readmission and speculates that such outcomes are likely more costly than providing good quality communication in the first instance:

*“ … if you don’t give the information to the patient (.) patient will be back in the hospital maybe in a months’ time (.) that means again you have to look after him (.) you have to spend more money looking after him then typing a few letters”*

## Discussion

### Key findings

The study identified a wide range of patient views and experiences on the topic of discharge letters. The majority valued receiving discharge letters, but many recognised that consideration of choice is important as not all patients may want to receive letters. It was felt that providing letters can alleviate anxiety supporting wellbeing, but participants suggested ways of enhancing the format and content of letters to increase their usefulness.

Participants’ experience of receiving discharge letters related to their recent episode of care was inconsistent; this is despite this being considered to be good practice in the UK [2, 4]. The study found that most (88%) participants indicated that they wanted to receive a copy of their recent discharge letter. Some participants recalled occasions where they had received letters and others where this had not happened. Participants appeared generally unsure about reasons for why such inconsistency occurs. Contextual factors such as time limitations on discharging physicians and differing hospital departmental discharge policies may be impacting upon this. The extent to which this applies to the wider NHS population needs further investigation.

A number of consequences of patients receiving letters (or not) were identified. Outcomes for patients receiving letters were generally conceptualised as beneficial: increased understanding and awareness of condition and next steps (e.g. treatment plan), reduced patient anxiety and improved patient well-being, letter acted as reminder/reference (e.g. for future appointments), improved letter quality as patients can amend inaccuracies, actioned follow ups as patients able to prompt these if necessary, and increased patient satisfaction. Participants generally emphasised that for outcomes to be positive, letters should be presented in a clear and accessible format that reflects the priorities and information needs of patients. Notably, no sample participant reported having seen their GP to have their letter explained and many participants felt that they understood all or enough of the letter for it to be useful to them or they were able to clarify unknown terms through internet searching or other resources. Impacts and outcomes of cases where participants *had not* received letters were generally conceptualised as negative: letter inaccuracies, “missed” follow ups, patients unable to fully remember title of diagnosis or recommendations, and patient confusion and anxiety at what occurred and what will happen next.

Although participants generally expressed preference to receive discharge letters, some expressed reservations or did not seem in favour of this practice (*N* = 6, 12%). Due to the qualitative nature of data and the relatively small sample size, it was not possible to formally quantify those who did not want to receive letters in terms of demographics or discharge characteristics. Contexts relating to such participants included: lack of interest in the information, having frequently attended hospital for the same issue, and not wanting to waste paper. Generally, participants’ opinions about receiving copies of letters were viewed as a matter of personal preference rather than specific to particular specialties or episodes of care. Participants felt patients should be offered a *choice* about when and whether they wish to receive a copy letter, a view supported by findings from past studies [8, 16, 19, 60]. Responses seemed to exhibit preference for “opt-out” letter receipt systems which would account for individuals who do not want letters whilst still allowing the majority of patients to receive letters. It was also noted that those who did not want to be exposed to the letters’ contents could dispose of the letter, pass it on to others (e.g. partner), or decide not to read the letter.

Many participants favoured receiving a copy of what is sent to the GP (31/50, 62%) but a few (7/50, 14%) did prefer the idea of a personalised letter. The remainder of participants either had no preference or wanted both or neither letter form. Notably, of the few who reported recent experience with receiving patient personalised letters, these were often described negatively. Those who favoured patient personalised letters appeared to be basing this on hypothesising the potential usefulness of such letters. Reasons for personalised letter preference tended to include reference to features which resonated with the *NICE* guidelines [1], other policies [2, 4, 59, 61, 62] and past works [8, 63] such as: lay explanations for jargon, minimising unexplained acronyms, an additional section for the patient (e.g. dietary advice), and a simple summary of results. Participants also suggested PALS and relevant contact information should be in the letter. Arguably, these are elements which could be integrated into the letters sent to GPs to ensure the letters are “patient friendly” whilst at the same time adhering to patient preferences for receiving copies; this would eradicate the need for the production of two separate discharge letters.

### Implications for practice

This research supports policies [1–4, 64] that all patients should be offered copies of letters between physicians. However, the study’s findings indicate inconsistent patient receipt of discharge letters; this suggests a need to review this guidance and its implementation. We suggest that the practice of patients receiving letters requires standardisation and auditing in order to increase policy uptake.

The study participants, in line with past studies [15–24, 65], showed high rates of preference for receiving letters. Sending patients direct copies of GP letters was not only favoured by many of the patients but could save time and resources through eradicating the need to produce two separate discharge letters (i.e. one for patient and one for GP); sending patients copies also adheres to good practice [2] and fits with the broader *NHS England* policy movement [66, 67] toward *shared-decision making* [68] and *patient-centred care* [69] (see also literature relating to shared-decision making and patient-centredness [70–75]). Copying patients into discharge letters about their episode of care enables self-management and self-efficacy; thus, empowering patients with such information may reduce the risk of important clinical information being overlooked (e.g. follow up blood tests). However, to increase clarity and usefulness, letters need to have a transparent format, precise instructions with comprehensible language, and clearly convey the information that this study has highlighted as being valued by patients. Importantly, the patients were not unanimous in their views and so systems need to be grounded by patient *choice.*

Participants were concerned about systems of letter receipt that required patients to make choices at the time of discharge when the patient may be fatigued or cognitively impaired (e.g. under effects of general anaesthesia *“you forget anaesthetic makes you forget things”)*. Participants suggested that they should be able to log preferences on their electronic health record; this would allow patients to “opt out” or change their mind for particular discharge events (e.g. repeat care episode or admission for same issue, bad news …) whilst still ensuring consistent practices of letter receipt. This system suggestion may be likened to the “Sharing Letters with Patients Policy” by *the Newcastle upon Tyne Hospitals NHS Foundation Trust* [59, 62].

### Strengths and limitations

The study was strengthened by the CL techniques used to analyse the data. This method allowed in depth consideration of the language used to describe discharge experiences; this afforded detailed analysis of meaning-making and how participants evaluated their discharge experiences. The corpus categorisation system coded texts for interview question and participant. This system allowed for rapid considerations of dispersion across interview questions and participants so that it could be ascertained whether a feature was dispersed across multiple participants or simply used multiple times by the same participant. Thus, the categorisation system partially controlled for over-representation of views of those who talked more during interviews.

The median time length between the hospital discharge and the interview with KW was 10 weeks (range 4–22 weeks). This may have affected the interviewee’s recall, and in the interim, some may have had further follow up (e.g. readmission, outpatient attendance …) with the hospital. The study was strengthened by the high availability of letters at interviews (43/50 or 86%) which likely reduced recall bias. However, the opportunity for participants to request to see their letter at interview may have incentivised some patients who had not previously seen their letter to take part which may have biased the sample. Nevertheless, showing participants their letters, if desired, was felt to be an important element of the protocol [25]; it reflects the project foundations which are to generate evidence to increase patient autonomy and empowerment, and facilitate patient-led and patient-centred care.

The study included a diverse sample of participants in terms of age, speciality of care and setting; this contrasts with many previous studies which tend to focus on single specialties such as Cardiology [76–78] and Oncology [15, 79–81]. However, much of the information about patient characteristics was missing. It appeared that ethnic minorities, low-literacy, and other minority and marginalised groups were under-represented in the sample; such individuals may feel differently about receiving letters to those sampled here. In light of this, future research should consider ways of enabling participation of these groups, such as recruiting patients through routes other than written materials (e.g. opportunistic recruitment through health and community services, recruitment drives at participating GP practices with members of the research team being present to promote the importance of the study …).

Episodes of inpatient discharge are over-represented in the data (88% of the participant sample); as the study sought to represent a range of discharge experiences this is a study weakness as findings cannot be assumed to apply to letters following outpatient attendances. This over-representation of inpatient discharges may have been due to the GPs misinterpreting the “discharge” element of the study criteria, GPs choosing to select inpatient letters as they represented key “successful” and “unsuccessful” exemplars, or because inpatient episodes are more likely to involve more significant clinical events and so this group of patients may have been more inclined to take part.

## Conclusions

The findings revealed insights into patients’ views of discharge letters which are vital for a continually progressive healthcare system which places patients and models of shared-decision making at its core. The majority of participants wanted to receive letters, but many also felt that it was important to allow patients *choice* about when they wish to receive letters (or not). They also expressed the importance of such letters being presented in a clear and accessible format that reflects the priorities and information needs of patients. Patients may not be receiving or being offered copies of letters consistently despite UK guidelines supporting this practice. This represents a possible missed opportunity to involve patients in their care and promote self-management in order to improve experiences and outcomes.

## Supplementary information

**Additional file 1.** Patient interview schedule.

**Additional file 2.** Summary of participant characteristics.

**Additional file 3.** Top 100 ranked keywords by “keyness” in patient corpus.

**Additional file 4.** Concordance samples for keywords discussed in the main manuscript.

**Additional file 5.** Table of word counts for corpus and sub-corpora.

## Data Availability

The datasets generated during the current study are not publicly available due to the sensitive and identifiable nature of the data. Despite names and other identifiers being removed, the in-depth nature of the interviews themselves may mean that participants can be identified from the full transcripts. For this reason, quotes and extracts only have been used in outputs and additional files. Further quotations are available from the corresponding author on reasonable request.

## Abbreviations

BNC: British National Corpus
CL: Corpus Linguistics
GP: General Practitioner (UK family or primary care physician)
NICE: National Institute for Health and Care Excellence
NHS: UK National Health Service
PALS: Patient Advice and Liaison Service
RQ: Research question
UK: United Kingdom
