# Supplementary material for: Adult patient perspectives on receiving hospital discharge letters: a corpus analysis of patient interviews
Source: BMC Health Serv Res. 2020 Jun 15;20:537. doi: 10.1186/s12913-020-05250-1 (PMC7294646; doi:10.1186/s12913-020-05250-1)
Supplement: Supplementary file 1 — Additional file 1. Patient interview schedule. [file 12913_2020_5250_MOESM1_ESM.docx]

**Patient interview schedule**

I: Interviewer (member of the research team) *Action points Q= Question

I: ***Q1: Please tell me about your experiences of receiving any form of written discharge communication? This can be either a direct copy of the letter sent to your GP or a discharge letter specifically addressed to yourself.***

***Q2: When you were discharged from hospital on DATE, what information were your given?***

****if patient able to be shown letter copy as per protocol, show patient their letter****

***Q3: How did you feel about the information you were given?***

***Q4: What written information would you like to be given or sent when being discharged from hospital and why?***

***Q5: Would you prefer to receive a direct copy of the letter sent to your GP or a separate letter specifically addressed to yourself?***

***Q6: Would you like to always be given this letter or would you prefer to choose each time you are discharged?***

***Q7: How do you think the process of patients receiving written discharge communication can be improved?***

***Q8: Is there anything else you would like to talk to me about today related to written discharge communication?***

*Discussion may continue in a relaxed conversational manner and researcher may ask additional questions related to anything else relevant mentioned by the patient.*
