# Supplementary material for: Adult patient perspectives on receiving hospital discharge letters: a corpus analysis of patient interviews
Source: BMC Health Serv Res. 2020 Jun 15;20:537. doi: 10.1186/s12913-020-05250-1 (PMC7294646; doi:10.1186/s12913-020-05250-1)
Supplement: Supplementary file 2 — Additional file 2. Summary of participant characteristics. [file 12913_2020_5250_MOESM2_ESM.docx]

*Summary of participant characteristics*

| **Demographics and characteristics** | **Respondent sample** | | |
| --- | --- | --- | --- |
|  | **Group** | **Frequency (%)** |  |
| Age | \| Unanswered \| \| --- \| \| 18-30 \| \| 31-40 \| \| 41-50 \| \| 51-60 \| \| 61-70 \| \| 71-80 \| \| 81-90 \| \| Total \| | \| 13 (26%) \| \| --- \| \| 1 (2%) \| \| 1 (2%) \| \| 10 (20%) \| \| 3 (6%) \| \| 10 (20%) \| \| 10 (20%) \| \| 2 (4%) \| \| 50 (100%) \| |  |
| Gender | Unanswered | 20 (40%) |  |
|  | Female | 16 (32%) |  |
|  | Male | 14 (28%) |  |
|  | Total | 50 (100%) |  |
| Ethnicity | \| Unanswered \| \| --- \| \| British \| \| English \| \| White \| \| White British \| \| Total \| | \| 19 (38%) \| \| --- \| \| 11 (22%) \| \| 5 (10%) \| \| 7 (14%) \| \| 8 (16%) \| \| 50 (100%) \| |  |
| Religion | \| Unanswered \| \| --- \| \| Church of England \| \| Catholic \| \| Christian (unspecified) \| \| None \| \| Total \| | \| 20 (40%) \| \| --- \| \| 18 (36%) \| \| 3 (6%) \| \| 4 (8%) \| \| 5 (10%) \| \| 50 (100%) \| |  |
| Highest education qualification | \| Unanswered \| \| --- \| \| GCSES/ O levels \| \| A Level \| \| NVQ \| \| Diploma or HND \| \| Undergraduate degree \| \| Teaching certificate \| \| Postgraduate degree \| \| Total \| | \| 30 (60%) \| \| --- \| \| 2 (4%) \| \| 4 (8%) \| \| 2 (4%) \| \| 2 (4%) \| \| 5 (10%) \| \| 3 (6%) \| \| 2 (4%) \| \| 50 (100%) \| |  |
| Income bracket (£) | \| Unanswered \| \| --- \| \| <12,000 \| \| 12 -18,000 \| \| 18 - 25,000 \| \| 25 - 40,000 \| \| 40,000+ \| \| Total \| | \| 32 (64%) \| \| --- \| \| 3 (6%) \| \| 3 (6%) \| \| 3 (6%) \| \| 5 (10%) \| \| 4 (8%) \| \| 50 (100%) \| |  |
| Registered GP practice region | \| Coventry \| \| --- \| \| Herefordshire \| \| North Warwickshire \| \| Rugby \| \| South Warwickshire \| \| Total \| | \| 12 (24%) \| \| --- \| \| 7 (14%) \| \| 3 (6%) \| \| 7 (14%) \| \| 21 (42%) \| \| 50 (100%) \| |  |
| Discharge speciality | \| Accident and Emergency (A&E) \| \| --- \| \| Acute internal Medicine \| \| Cardiology \| \| Colorectal Surgery \| \| Endocrinology \| \| General Medicine \| \| General Surgery \| \| Neurosurgery \| \| Ophthalmology \| \| Plastic Surgery \| \| Renal/Nephrology \| \| Respiratory \| \| Stroke Medicine \| \| Trauma & Orthopaedics \| \| Urology \| \| Total \| | \| 6 (12%) \| \| --- \| \| 1 (2%) \| \| 4 (8%) \| \| 1 (2%) \| \| 2 (4%) \| \| 4 (8%) \| \| 7 (14%) \| \| 1 (2%) \| \| 1 (2%) \| \| 1 (2%) \| \| 1 (2%) \| \| 6 (12%) \| \| 1 (2%) \| \| 8 (16%) \| \| 6 (12%) \| \| 50 (100%) \| |  |
| Type of care episode/admission | \| Inpatient \| \| --- \| \| Outpatient \| \| Other (e.g. A & E visit no admission) \| \| Total \| | \| 44 (88%) \| \| --- \| \| 3 (6%) \| \| 3 (6%) \| \| 50 (100%) \| |  |
