# Supplementary material for: Adult patient perspectives on receiving hospital discharge letters: a corpus analysis of patient interviews
Source: BMC Health Serv Res. 2020 Jun 15;20:537. doi: 10.1186/s12913-020-05250-1 (PMC7294646; doi:10.1186/s12913-020-05250-1)
Supplement: Supplementary file 3 — Additional file 3. Top 100 ranked keywords by “keyness” in patient corpus. [file 12913_2020_5250_MOESM3_ESM.docx]

| **Rank** | **No. of hits** | **Keyness (LL)** | **Keyword** | **Rank** | **No. of hits** | **Keyness (LL)** | **Keyword** | **Rank** | **No. of hits** | **Keyness (LL)** | **Keyword** | **Rank** | **No. of hits** | **Keyness (LL)** | **Keyword** |
| --- | --- | --- | --- | --- | --- | --- | --- | --- | --- | --- | --- | --- | --- | --- | --- |
| **1** | 612 | 4291.52 | hospital | **26** | 1913 | 987.84 | they | **51** | 93 | 548.26 | appointment | **76** | 572 | 369.89 | about |
| **2** | 400 | 3954.06 | discharge | **27** | 209 | 921.68 | given | **52** | 78 | 523.5 | communication | **77** | 453 | 369.5 | mean |
| **3** | 6861 | 3866.16 | i | **28** | 1957 | 904.38 | of | **53** | 825 | 516.44 | this | **78** | 143 | 366.7 | understand |
| **4** | 3967 | 3335.26 | to | **29** | 1613 | 861.72 | so | **54** | 63 | 514.53 | summary | **79** | 269 | 350.78 | has |
| **5** | 4838 | 3321.96 | and | **30** | 1404 | 810.56 | know | **55** | 792 | 494.17 | if | **80** | 1926 | 350.12 | t |
| **6** | 971 | 3239.42 | um | **31** | 116 | 795.17 | surgery | **56** | 2397 | 493.84 | a | **81** | 409 | 347.61 | see |
| **7** | 5000 | 3174.57 | the | **32** | 1027 | 790.04 | think | **57** | 103 | 491.17 | follow | **82** | 192 | 347.19 | uh |
| **8** | 465 | 3160.67 | letter | **33** | 129 | 786.87 | copy | **58** | 1538 | 483.74 | in | **83** | 77 | 346.46 | letters |
| **9** | 491 | 2996.21 | its | **34** | 867 | 783.44 | my | **59** | 80 | 478.56 | operation | **84** | 49 | 330.53 | surgeon |
| **10** | 275 | 2441.04 | gp | **35** | 78 | 781.35 | ive | **60** | 74 | 478.48 | consultant | **85** | 270 | 324.98 | will |
| **11** | 372 | 2426.71 | information | **36** | 1234 | 759.28 | what | **61** | 50 | 477.84 | aftercare | **86** | 58 | 319.63 | results |
| **12** | 1917 | 2364.36 | have | **37** | 545 | 757.4 | been | **62** | 57 | 477.81 | transcribed | **87** | 59 | 316.75 | tests |
| **13** | 350 | 2128.04 | doctor | **38** | 88 | 753.69 | ward | **63** | 61 | 462.14 | laughs | **88** | 926 | 314.18 | there |
| **14** | 1019 | 1953.98 | because | **39** | 249 | 733.02 | am | **64** | 995 | 455.07 | on | **89** | 88 | 311.55 | waiting |
| **15** | 2214 | 1625.94 | was | **40** | 717 | 730.22 | as | **65** | 533 | 443.37 | said | **90** | 52 | 308.22 | paperwork |
| **16** | 1044 | 1579.87 | me | **41** | 859 | 711.77 | with | **66** | 428 | 418.04 | time | **91** | 234 | 306.94 | anything |
| **17** | 174 | 1537.5 | im | **42** | 112 | 696.44 | nurse | **67** | 41 | 401.26 | whats | **92** | 50 | 305.82 | received |
| **18** | 3324 | 1527.67 | that | **43** | 1353 | 686.69 | is | **68** | 182 | 399.29 | told | **93** | 537 | 301.16 | when |
| **19** | 152 | 1485.56 | discharged | **44** | 75 | 685.67 | cant | **69** | 57 | 398.37 | nurses | **94** | 617 | 298.79 | or |
| **20** | 971 | 1457.51 | had | **45** | 982 | 625.2 | be | **70** | 3062 | 393.54 | you | **95** | 241 | 296.54 | should |
| **21** | 177 | 1437.48 | patient | **46** | 487 | 621.4 | say | **71** | 452 | 389.97 | going | **96** | 73 | 286.63 | blood |
| **22** | 151 | 1195.41 | medication | **47** | 1447 | 615.12 | but | **72** | 413 | 387.42 | very | **97** | 299 | 264.52 | which |
| **23** | 779 | 1151.55 | would | **48** | 96 | 583.8 | medical | **73** | 418 | 384.6 | from | **98** | 38 | 263.23 | reads |
| **24** | 173 | 1147.95 | doctors | **49** | 960 | 581.78 | for | **74** | 64 | 372.61 | tablets | **99** | 119 | 262 | happened |
| **25** | 924 | 1055.58 | are | **50** | 73 | 548.96 | patients | **75** | 66 | 370.3 | treatment | **100** | 3198 | 259.34 | it |

*Top 100 ranked keywords by “keyness” (log-likelihood (LL)) (p<.05) in patient corpus*
