# Supplementary material for: Adult patient perspectives on receiving hospital discharge letters: a corpus analysis of patient interviews
Source: BMC Health Serv Res. 2020 Jun 15;20:537. doi: 10.1186/s12913-020-05250-1 (PMC7294646; doi:10.1186/s12913-020-05250-1)
Supplement: Supplementary file 4 — Additional file 4. Concordance samples for keywords discussed in the main manuscript. [file 12913_2020_5250_MOESM4_ESM.docx]

**Concordance samples for keywords discussed in the main manuscript**

*Sample of 10 random mixed lines for [SAY] and [TELL]*

| handwritten letter to me um just | **saying** | I was okay to go and that was it really (.) so |
| --- | --- | --- |
| because I was uh had a letter to | **say** | to go their clinic and when I got there there was |
| the actual piece of paper doesn’t | **tell** | you much at all got a bit more when I was |
| still don’t know exactly what was | **told** | the doctors I know they will show me exactly what |
| ﻿Um (.) well they just um | **told** | me that it was me breathing (.) I was breathing very |
| to have that (.) and she | **said** | apart from finding out that there is no cancer there |
| [LETTER] one (.) they have | **said** | I have got a [CONDITION] which I haven’t (.) its |
| other things going on and they did | **tell** | that he couldn’t come around to see me because |
| to the GP and what they have | **said** | in the past erm you shouldn’t be on it no longer |
| point I don’t know when they just | **said** | they will be in touch and that was it that’s all I |

*Sample of 10 lines for lemma [FEEL] in Q3 from 10 different patients*

| recommend you should do (.) I did | **feel** | a bit uneasy (.) they had obviously ruled out |
| --- | --- | --- |
| medication or review so that’s where I | **feel** | there was an error definitely (.) so it wasn’t |
| well so it just wasn’t organised and I did | **feel** | like they just completely wiped their hands |
| so I know no it was a good summary I | **felt** | it was a good summary (.) that I could |
| it (.) it wont go nowhere (.) but I | **felt** | very strongly this time that the (.) it was very |
| feeling uh I think it adds to your | **feeling** | of vulnerability because you cant have a full |
| go through my mind of you know | **feeling** | worried about going under you know so it |
| plates in my leg at the time so I | **feel** | that process and the interaction has |
| ﻿ I did I felt alright about it um I | **felt** | a bit that why did they put this instead of |
| positive step forward I definitely | **felt** | good about it yeah and there was some trust |

*Sample of 10 random concordance lines for lemmatised form of “information”*

| um may feel that too much | **information** | is scary but the thing is you can prepare |
| --- | --- | --- |
| medication so I think detailed | **information** | from day 1 to the final letter should be given |
| they provide you loads of | **information** | but you can’t remember everything and so |
| looking forward to getting detailed | **information** | as to the extent to which the disease has |
| situation and you need to have the | **information** | in the discharge it needs to be clear to |
| the end because uh (.) that has the | **information** | you need (.) it tells you what is going to be |
| ﻿To be honest I have (.) the | **information** | that I received was ample was sufficient for |
| the near future just more practical | **information** | on um on your wellbeing if you like how |
| should give you all the relevant | **information** | verbally and by letter (.) I know it probably |
| bit more information and better | **information** | (.) |

*All lines for “personalised” with the Q5 sub-corpus*

| details the specific patient (.) the | **personalised** | information these are the sort of |
| --- | --- | --- |
| had done just a little bit more | **personalised** | letter but on the other hand I would |
| I don’t really mind though a | **personalised** | one because I don’t know the person |
| ﻿ it would be nice to have a | **personalised** | one because obviously what they |
| go that far but to me that’s what a | **personalised** | one would be best (.) you know its |
| would be going too far to ask for a | **personalised** | (.) bit to what you need to look out |
| ﻿I think I would have liked a | **personalised** | is always nice to have |
| ﻿yeah I think a | **personalised** | letter is but something like this |

*Sample of 10 lines for 10 different patients for “copy” in Q5 sub-corpus*

| ﻿I would just like a direct | **copy** | of what goes to the GP receives (.) I don’t |
| --- | --- | --- |
| ﻿Well I’m quite happy to have a | **copy** | of the letter sent to the GP (.) I’m not |
| just because he is a GP (.) I like the | **copy** | that I have which is corresponds with what |
| but for me personally yeah direct | **copy** |  |
| tells me what I want to know (.) and a | **copy** | of that would be fine (.) Yeah (.) it possibly |
| ﻿no I think just have a | **copy** | of what goes to the GP probably just |
| but I would have been expecting a | **copy** | follow up from the doctor just to explain |
| ﻿um just a | **copy** | of the letter to the GP would be absolutely |
| and I think yes we should have a | **copy** | of things sent to the doctor but you know |
| from [NAME] I think I like to see a | **copy** | of the GP letter (.) um yeah this erm he |

*Sample of 10 lines for lemmatised form of “understand” in Q5 sub-corpus*

| what they write to a GP I might not | **understand** | as well as a letter specifically tailored to |
| --- | --- | --- |
| into anything that a lay person can | **understand** | and you know I was quite surprised |
| have got medical knowledge so I would | **understand** | it and I would like to see exactly what |
| ﻿I don’t | **understand** | why there should be a difference in what |
| take it with to the GP and say I don’t | **understand** | the third paragraph here that is talking |
| think (.) anymore of it (.) because I | **understand** | all that so that would be it as far as I am |
| and I read it but whether I would have | **understand** | it I don’t know and probably not I would |
| in plain English so that everyone can | **understand** | it and that’s how it should look |
| in there that I don’t (.) I wouldnt | **understand** | and I think (.) its not necessary (.) for |
| like a patient one (.) because I would | **understand** | it more maybe than one that goes to the |

*Sample of 10 lines for 10 different patients for “opt”*

| think it would work better as an | **opt** | in that patients were given a letter (.) I |
| --- | --- | --- |
| (.) rather than if its the addition | **opt** | out yeah |
| this occasion (.) I think probably an | **opt** | out to be honest with you (.) I think the |
| yeah (.) um (.) I think the first time | **opt** | in (.) I think they should have a discharge |
| they have been doing I mean you could | **opt** | in to it anyway (.) yeah without having to |
| (.) I think well I guess its good (.) | **opt** | out really from my point of view (.) because |
| the tests were (.) why why should I | **opt** | in for that (.) it should be automatic it |
| if you need to erm I think opt in | **opt** | out would be good (.) because obviously if |
| those who don’t want it they just | **opt** | out (.) |
| I don’t know but yeah I think an | **opt** | out (.) I think it should be a standard thing |

*Random sample of 10 lines for lemma “automatic”*

| why should I opt in for that (.) it should be | **automatic** | it should just be protocol (.) its |
| --- | --- | --- |
| think its a good thing and for that to be | **automatic** | as this obviously is (.) I mean |
| else to go astray whereas if you do it | **automatically** | then I think you (.) its probably |
| on the individual so rather than it being | **automatic** | which I favour some people |
| to have one every time yup (.) well | **automatic** | yes |
| ﻿ no always give it (.) have a letter (.) yes | **automatic** | yes (.) yeah for me personally |
| ﻿I think probably just | **automatically** | I think it should be their policy |
| would not presume to open it (.) so yes | **automatic** | (.) ohhh (.) okay so I know they |
| bit I had to ask them for (.) they didn’t | **automatically** | give you that (.) and he said |
| I would like a letter then it would | **automatically** | come through and you |

*Sample of 10 random concordance lines for lemma “discharge”*

| so not very positive experience of that | **discharge** | either (.) I read the information when I got |
| --- | --- | --- |
| take the hospital visit as I was being | **discharged** | I was handed a letter in an envelope |
| they going to be ready where is the | **discharge** | letter because we didn’t know what the |
| I really didn’t understand what the first | **discharge** | letter to me was useful for I can |
| in and out a few times so (.) received | **discharge** | papers for them (.) um I was taken in |
| and it says each hospital has its own | **discharge** | policy I am just wondering why (.) why |
| a letter from the hospital when I was | **discharged** | um which basically said which medication |
| it wasn’t ideal but the discharge the | **discharge** | was a bit hit and miss really and |
| that was given (.) and the way I was | **discharged** | was very poorly done (.) the first time I |
| uh usually as a rule when you get | **discharged** | you don’t get a lot of information (.) |

*All lines for “medical” with the Q5 sub-corpus*

| judgement on that (.) so I think in the | **medical** | case um the technical stuff Is there to give to the |
| --- | --- | --- |
| the full names of them It’s sort of | **medical** | jargon isn’t it (.) every profession has its own list |
| ﻿ Yeah probably because I have got | **medical** | knowledge so I would understand it and I would |
| with me they didn’t tell me what the | **medical** | name of it was and I could obviously feel that I |
| so (.) because I don’t there is a lot of | **medical** | stuff in there that I don’t (.) I wouldnt |
| would be difficult because it would be | **medical** | terms and they wouldnt have a clue what it was |
| but I have taken a lot of interest in | **medical** | things some people might find it a bit daunting I |

*Sample of lines where patients articulate they cannot “think” of anything else to add for Q8*

| ﻿I don’t think so no I cant | **think** | of **anything** else |
| --- | --- | --- |
| so I think I’m alright really (.) Um I cant | **think** | of **anything** really |
| I think we have covered it I mean if I | **think** | of **anything** I will give you a bell |
| ﻿No I cant | **think** | of **anything** (.) to do with this current sort of |
| ﻿I cant | **think** | of **anything** (.) no nothing (.) can’t think of |
| had that long off before um (.) but I cant | **think** | of **anything** else I actually thought at the time or |

*Illustrative sample of 10 lines for lemmas “test” and “result”*

| comprehensive um (.) they even gave | **results** | and treatment and management *gestures to |
| --- | --- | --- |
| a very detailed letter about all the | **tests** | they did and what they found (.) I went into |
| (.) no she had to wait for same for the | **results** | of the tests for lots of the stuff that they took out |
| was more you know they have all the | **tests** | and everything but I’ve not gotten anything (.) I |
| (.) that I needed a kidney function | **test** | which I knew from a discharge in [DATE] and I’ve |
| was there including the blood | **results** | as well so yeah (.) |
| to wait a few weeks to find out the | **result** | was not a stroke which I was on the edge like this |
| with discharge if you have had any | **tests** | you need the results of those tests if they found |
| with what you went in for what they | **tested** | you for (.) what the prognosis is and what the |
| what was it for and what was the | **results** | I didn’t know (.) so I was ignored most of the time |
