# Supplementary material for: Adult patient perspectives on receiving hospital discharge letters: a corpus analysis of patient interviews
Source: BMC Health Serv Res. 2020 Jun 15;20:537. doi: 10.1186/s12913-020-05250-1 (PMC7294646; doi:10.1186/s12913-020-05250-1)
Supplement: Supplementary file 5 — Additional file 5. Table of word counts for corpus and sub-corpora. [file 12913_2020_5250_MOESM5_ESM.docx]

**Table of word counts for corpus and sub-corpora**

|  | **Patient interview corpus** | | | | | | | |
| --- | --- | --- | --- | --- | --- | --- | --- | --- |
| **Sub-corpora** | Q1 | Q2 | Q3 | Q4 | Q5 | Q6 | Q7 | Q8 |
| **Word counts** | 18,468 | 14,126 | 19,746 | 19,464 | 9,748 | 12,909 | 19,250 | 21,926 |
| **Total word count** | 135, 637 | | | | | | | |
